# EEG-Validated Photobiomodulation Treatment of Dementia—Case Study

**DOI:** 10.3390/s22197555

**Published:** 2022-10-05

**Authors:** Miroslav Vrankic, Saša Vlahinić, Zoran Šverko, Ivan Markovinović

**Affiliations:** Department of Automation and Electronics, Faculty of Engineering, University of Rijeka, 51000 Rijeka, Croatia

**Keywords:** photobiomodulation, near-infrared stimulation, power spectrum analysis, brain connectivity analysis

## Abstract

In this article, we perform a case study of the impact of photobiomodulation (*PBM*) on brain power spectrum and connectivity in an elderly person with a Self Administered Gerocognitive Exam (*SAGE*) score indicating probable memory and thinking disorder. First, we designed and realized the prototype of a near-infrared (*NIR*) device for *PBM*. Analysing the alpha band of the power spectrum, we found a positive long-term effect in nine out of sixteen electrodes in the eyes-open condition (*OE*) and in twelve out of sixteen electrodes in the eyes-closed condition (*CE*), while in the theta band, a positive long-term effect was found in nine out of sixteen electrodes for *OE* and seven out of sixteen electrodes for *CE*. When considering the theta-alpha ratio (*TAR*), the positive long-term effect is found on thirteen of sixteen electrodes for *OE* and on fourteen of sixteen electrodes for *CE*. A connectivity analysis using the imaginary component of the complex Pearson correlation coefficient (*imCPCC*) was also performed, and a global efficiency measure based on connectivity matrices with thresholds was calculated. The global efficiency calculated for the long-term effect was higher than before stimulation by a factor of 5.24 for the *OE* condition and by a factor of 1.25 for the *CE* condition. This case study suggests that *PBM* could have positive effects on improving desired brain activity, measured as improvement in power spectrum and connectivity measures in theta and alpha bands, for elderly people with memory and thinking disorders.

## 1. Introduction

The human brain is a system of neurons ($10^{11}$) connected by a multitude of synaptic connections ($10^{14}$). Today, research to understand the human brain complex is going in several directions and is conducted by scientists from different fields such as social sciences, biology, information technology, and image processing [1].

Brain damage and disease affect the development of various diseases in people of all ages. Degenerative diseases such as dementia and Parkinson’s disease, as well as strokes and headaches, are common neurological conditions in elderly patients [2]. In this article, we will focus on dementia.

### 1.1. Causes and Symptoms

Dementia refers to a wide range of medical conditions that typically affect elderly persons, including memory impairments and various other decreases of cognitive functioning. Those conditions are triggered by abnormal brain changes which are not a part of normal aging. The most common cause of dementia is Alzheimer’s disease, estimated to contribute by 60% to 80% to the total number of dementia cases [3]. Another type is vascular dementia which is usually connected to a stroke and issues with blood vessels in the brain. This type of dementia accounts for up to 10% of dementia cases. Lewy bodies dementia is the third type of dementia in order of occurrence amounting to an estimated of 5% to 10% of all dementia cases [4]. It is related to abnormal formations in the brain called Levy bodies whose main component is the alpha-synuclein protein. Some other types and causes of dementia are Parkinson’s disease dementia [5], Frontotemporal Dementia [6], Huntington’s Disease [7], and Creutzfeldt-Jakob Disease [8]. In case of mixed dementia there are multiple types of dementia present, such as a combination of Alzheimer’s disease and vascular dementia. Although most types of dementia are progressive and non-reversible there are some which are reversible such as dementia caused by vitamin deficiencies or thyroid problems. Dementia affects many aspects of a person’s life and the symptoms can be quite different, also depending on the type of dementia. Some of the symptoms are memory problems, mood and personality changes, trouble performing simple everyday tasks, losing things, poor planning, difficulty with speaking or understanding speech, problems thinking clearly and making decisions, difficulty with recognizing familiar sights and sounds, problems with walking and balance.

### 1.2. Diagnostics

Diagnostics of dementia are based on a set of cognitive tests combined with a physical exam, laboratory tests and taking a patient’s history regarding the onset of symptoms, other medical issues and medications being used. The cognitive tests assess various capabilities such as short-term and long-term memory, simple problem solving, attention, orientation in time, and language skills [9]. Additionally, brain scans such as Magnetic Resonance Imaging (*MRI*) and Computed tomography (*CT*) can be used to confirm a diagnosis of dementia.

Electroencephalography (*EEG*) is also a widely used method in diagnosing dementia [10]. It was shown that Alzheimer’s disease (*AD*) causes the power spectrum of *EEG* signals to be shifted towards lower frequencies. It is important to note that normal aging also causes a decrease of some spectral components of *EEG* signals such as alpha activity in temporal regions [11]. However, in case of *AD* the changes are much more severe and there is an overall decrease of a mean frequency of the *EEG* signals, decrease of alpha and beta components, and increase of delta and theta components [12].

It was shown that various types of dementia cause reduced interconnectivity between various cortical regions. However, this reduced interconnectivy is not the same for all the frequencies. Rather, it is dominant in higher frequency bands. A measure showing that is EEG coherence which is decreased for both close and distant channels for alpha and beta bands for various types of dementia [13]. This reduced coherence at higher frequencies is an influence of cognitive impairment.

Similarly, in [14], it was shown that the EEG features called epoch-based entropy (a measure of signal complexity) and bump modeling (a measure of synchrony) were sufficient for efficient classification between patients with subjective cognitive impairment (*SCI*) and possible *AD* patients (accuracy 91.6%) and with an accuracy of 81.8% to 88.8% for classification of *SCI*, possible *AD* and other patients. In [15], the classifier achieved almost 90% accuracy in classifying patients with mild cognitive impairment (*MCI*) and normal individuals. It was also found that selecting features from a combined set of power and coherence features produced better results than using each feature individually.

In [16], 26 patients diagnosed with AD, 53 MCI patients and 191 cognitively normal healthy participants were included in the study. In comparison between the control group and the AD group, an increase in spectral power and coherence at slower frequencies (delta and theta bands) was observed. In addition, the AD group showed a significant decrease in spectral and coherence analysis in the alpha band (consistent with the same effect in normal ageing). Furthermore, they conclude that the ratio of theta to alpha (TAR) shows the largest and most significant differences between the AD group and the controls [16].

The functional dysregulation of the default mode network that occurs with the progression of *AD* is often associated with a resting brain state similar to Alpha activity in the occipital area when the eyes are closed, as reported in [17,18].

A more comprehensive measure which takes into account both linear and nonlinear dependencies of various channels is called Mutual Information (*MI*). It was shown that mutual information between distant electrodes is decreased in patients with *AD* which can be explained by a reduced information transmission among various cortical regions [19].

Furthermore, in [20], Alzheimer’s dementia was analyzed using Global Field Synchronization (*GFS*) and patients showed a decrease in *GFS* values in the alpha, beta and gamma bands and an increase in *GFS* values in the delta band.

In [21], magnetoencephalography (*MEG*), connectivity analyses were performed with 18 *AD* patients and 18 patients in the control group, and *AD* patients showed a decrease in the mean phase lag index (*PLI*) value in the lower alpha and beta bands. In addition, both clustering coefficient and path length were decreased in *AD* patients.

### 1.3. Treatment of AD

Currently, the U.S. Food and Drug Administration (*FDA*) has approved several drugs (donepezil, the rivastigmine patch, and a combination drug of memantine and donepezil) for the treatment of moderate to severe Alzheimer’s disease [22]. At the same time, research is being conducted that is exploring a variety of options to not only treat the symptoms, but also to address the underlying disease processes. The possibility of modulating, facilitating, or interrupting this electrical activity of the brain is very appealing; it can help bring about temporary or reasonably permanent desirable changes in the brain [23]. Currently, various types of brain stimulation are being investigated, such as transcranial direct current stimulation (*tDCS*) [24], transcranial magnetic stimulation (*TMS*) [25], deep brain stimulation (*DBS*) [26], photobiomodulation (*PBM*) [17], etc. Although not yet used for *AD*, the alternative method of acupuncture to modulate brain activity is worth mentioning [27]. In this article, we focus on *PBM*.

### 1.4. Photobiomodulation (PBM)

**Figure 1:**
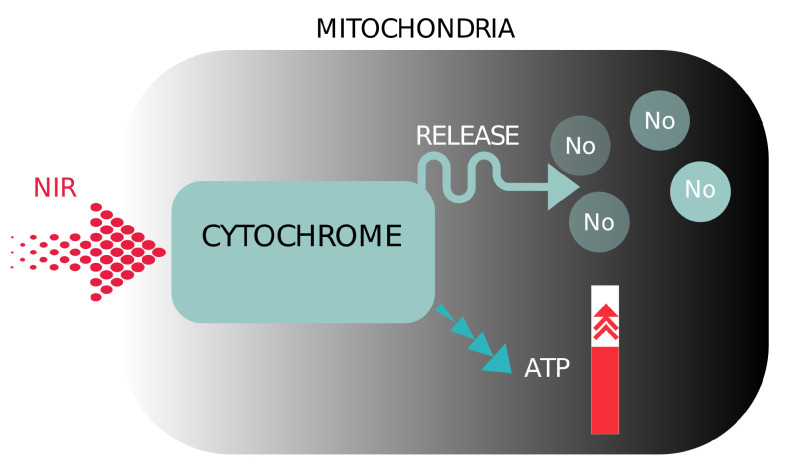
Illustration of the schematic representation of the mechanisms of photobiomodulation near infrared light.

Photobiomodulation (*PBM*) refers to the therapeutic use of red or near-infrared light to stimulate inflammation, relieve pain, promote healing, and prevent tissue death [17]. The photons of a certain energy or the light of a certain wavelength are able to stimulate a photochemical reaction by being absorbed by the photoaccepting chemical substances in the cell [18]. Different wavelengths can be used, but red and near-infrared (*NIR*) light with wavelengths from 600 to 1100 nm are most commonly used because of their greater penetration depth. With red and *NIR* light, the photoacceptor is cytochrome c oxidase, an enzyme in mitochondria involved in the fourth phase of the mitochondrial respiratory chain [18,28]. When *PBM* is used with red and *NIR* light, mitochondria in cells produce more *ATP* (Anti-inflammatroy process), which consequently leads to more energy for the cell. In addition, PBM produces reactive oxygen species, which play an important role in cell healing, signaling, and stimulation of gene transcription by transcription factors and nuclear factors [18,29]. Another benefit of PBM is the release of nitric oxide from the mitochondria, allowing more oxygen to take its place and generate more energy [18], see Figure 1.

Several studies have already used *PBM*. In [30], participants took part in 20 min of active transcranial photobiomodulation (*tPBM*) twice at least one weekend apart. The authors in [30] analyzed the power spectrum and connectivity analysis in all frequency bands.

In addition, the influence of transcranial infrared laser stimulation on the power spectrum was analyzed in [31]. The *TILS* was performed once a week for 5 weeks with a laser power density of 250 mW/cm${}^{2}$.

In [32], the effect of near-infrared photobiomodulation therapy was studied in patients with mild to moderate dementia or possible Alzheimer’s disease diagnosed with the Mini-Mental State Exam (*MMSE*). Patients were treated weekly in the clinic with a transcranial intranasal *PBM* device and daily at home with an intranasal device as part of a protocol. After 12 weeks of *PBM*, significant improvement in *MMSE* observation and improvement in function, less anxiety, less anger, wandering, and better sleep were noted.

In the study presented in [33], the effect of *tPBM* on default mode network connectivity was confirmed using functional magnetic resonance imaging.

## 2. Materials and Methods

### 2.1. Near Infra Red (*NIR*) Device Prototype

When selecting the *LEDs*, care was taken to ensure that the diodes radiate in the infrared range (*IR*), since it is at these wavelengths that light penetrates deepest into tissue and bone. In addition, a power of 1W was required to achieve greater reliability (due to operation at powers lower than the rated power). *LEDs* with the following properties were purchased:

- 1 W, 810 nm; *DC IF*: 700 mA; *DC UF*: typical: 1.6–2.2 V; beam angle 120–140${}^{\circ }$.

The control module is based on heltec-wifi-kit-32 development module and ESP32 microprocessor (dual-core 32-bit *MCU* + *ULP* cores; Wi-Fi, Bluetooth 2.4 GHz *PCB* antenna; 0.96-inch 128∗64 dot matrix *OLED* display; *LED PWM*, up to 16 channels; memory: 448KB *ROM*, 520 KB *SRAM*, 16 KB *SRAM RTC*, 4 MB *SPI FLASH*; Micro *USB* interface). A step-down buck converter with 9–36 V input and 5 V output and 12–24 V power supply was used to power the development module.

**Figure 2:**
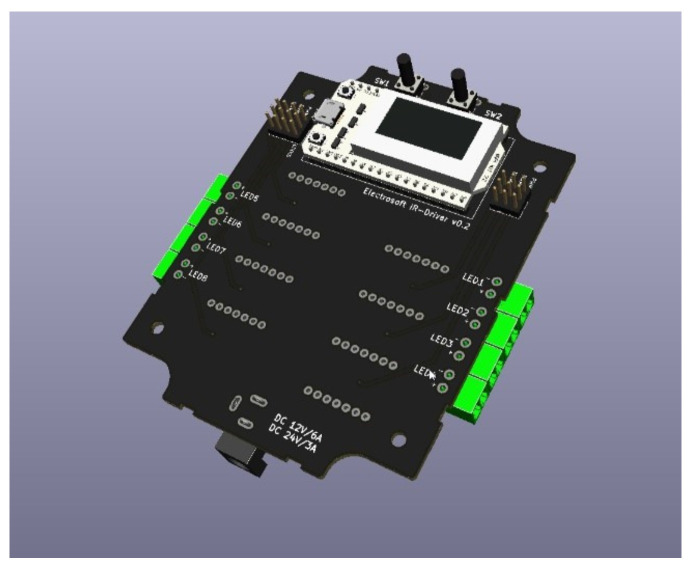
The *3D* model of the control module.

Four output channels *LED PWM* were used to power 4 clusters of 3 *LEDs* each, with the possibility of expansion to 8 channels. PT4115 3 W/700 mA max constant current *LED* driver was used as *IR LED* driver. The *3D* model of the control module is shown in the Figure 2 below.

Output power of the device can be changed in four levels, indicated by a bar graph and modulation with a rectangular signal of frequency 8 Hz, 10 Hz, 12 Hz, 20 Hz and 40 Hz.

Optical power measurements were made with a Newport 843-R instrument, accuracy ±0.25% of full scale. The diameter of the sensor used is 10 mm and the area is 0.79 cm${}^{2}$. The following Table 1 shows the mean value of measurements for *LEDs*, power (*P*) in mW and power density ($P_{g}$) in mW/cm${}^{2}$, for 3 diodes in a cluster. Square modulation frequencies of 10 Hz and 40 Hz and continuous generation (*DC*) were tested.

To reduce interference when recording *EEG* signals, two changes were also made: power is supplied by a battery and a two-wire shielded (and grounded) cable is used.

**Figure 3:**
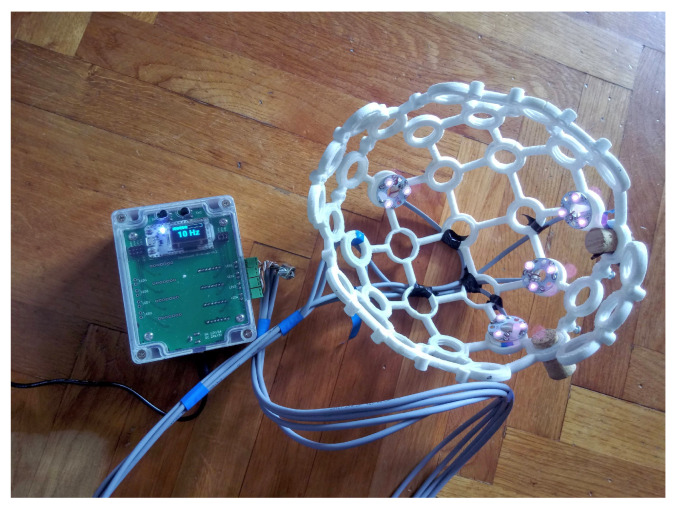
The cluster of *IR LEDs* mounted on a *3D*-printed holder (headset) [34] and a prototype produced.

A *3D*-printed *EEG* headset [34] was used to hold the *IR LED*. The headset used was selected based on the available *CAD* files and the ability to flexibly place the cluster of *IR LEDs*, see Figure 3.

One cluster of *IR LEDs* is located between P3/P7, another between P4/P8, and two more clusters are located above electrodes Fz and Pz.

### 2.2. Stimulation Protocol

Stimulations were performed over a period of 35 days. The stimulation frequency was set at 10 Hz. During the first week of stimulation, three sessions were performed at power level 2, whereas during the remainder of the period, five sessions per week were performed at power level 3.

### 2.3. EEG Acquisition

*EEG* recordings were made with sixteen Brain Vision actiCAP active electrodes and the Brain Vision V-Amp amplifier with a resolution of 24 bits or 48.9 nV and a sampling rate of 512 Hz. The ground electrode was located on the right mastoid and the reference electrode on the left mastoid. *EEG* signals were recorded at positions Fp1, Fp2, F3, Fz, F4, T7, C3, Cz, C4, T8, P3, Pz, P4, O1, Oz, and O2 of the International Standard 10−20 for electrode placement. An impedance check was performed using the Brain Vision Recorder, before the measurements and was less than 5 kΩ. The study design was implemented in OpenVibe software, and EEGLab Toolbox was used to import the data into Matlab.

### 2.4. Description of the Pilot Study Design

**Figure 4:**
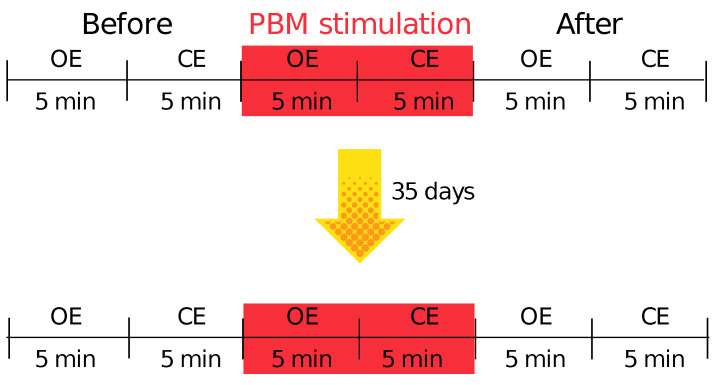
Schematic representation of the study design.

The measurement protocol consisted of the following parts: 10 minutes of *EEG* signal recording before stimulation (measurement interval marked “Before”) in two conditions with eyes open (*OE*) and eyes closed (*CE*) for 5 minutes each. Then *EEG* signals were recorded during photobiomodulation (measurement interval marked with “Stimulation”) and 10 minutes after photobiomodulation treatment (measurement interval marked with “After”). A schematic representation of the study design can be found in Figure 4.

**Figure 5:**
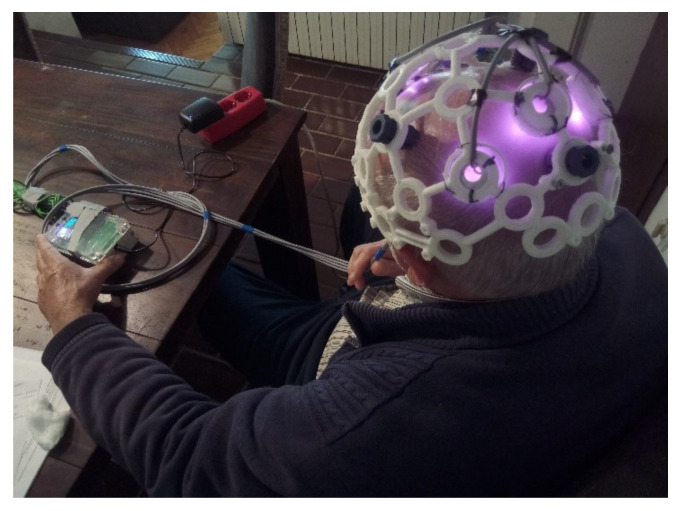
Schematic representation of the study design.

The prototype tests were performed on one subject: an elderly person (84 years old). Before starting the experiment, the subject was tested with the *SAGE* test. *SAGE* is a short questionnaire for self-assessment of cognitive status with the aim of detecting mild cognitive impairment (*MCI*) and early signs of dementia. The questionnaire takes an average of 15 minutes to complete. The maximum score is 22, and a score of 17 and above is considered normal (individuals with this score are within the normal range). Individuals with scores of 15 and 16 most likely suffering from a milder memory or thinking disorder. Individuals with a score of 14 or less are most likely to suffer from a more severe memory or thinking disorder [35]. In the first test with the questionnaire *SAGE*, the subject scored 16. See Figure 5 for the treatment of PBM stimulation.

### 2.5. Offline Preprocessing

**Figure 6:**
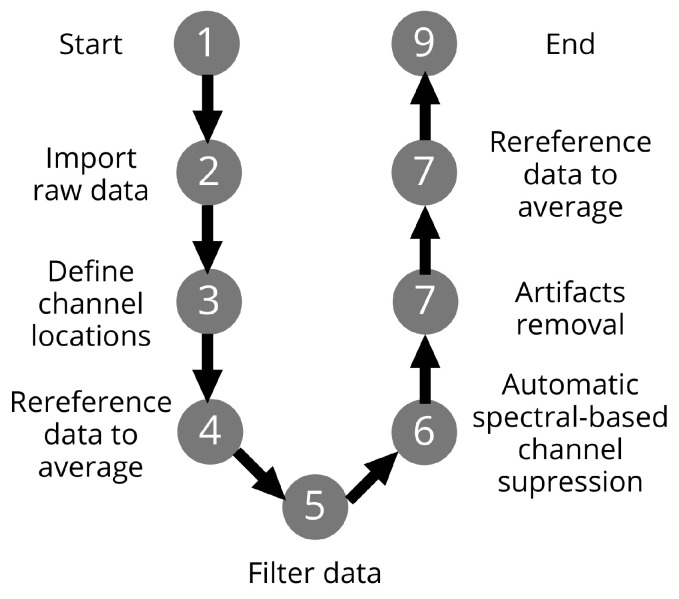
The illustration of the offline preprocessing steps.

The steps of offline preprocessing of raw EEG signals are shown in the flowchart, see Figure 6.

The EEGLab toolbox was used to import the raw *EEG* data into Matlab. In addition, channel positions were determined according to the international 10–20 standard. Furthermore, the data were referenced to the average and filtered to the desired frequency bands. Automatic spectral channel suppression (z=5) was performed using the EEGLab “*pop-rejchan*” function. Moreover, artifacts were removed using the EEGLab plugin IClabel. Thresholds to remove components were chosen that were greater than or equal to 0.9 for artifacts and less than or equal to 0.05 for brain activity. At the end, the data were referenced to the average.

### 2.6. Analyses

#### 2.6.1. Power Spectrum Analysis

Frequency analysis was performed by Fast Fourier transform (*FFT*) on time segments of length 1 s (512 samples) using the Hamming window function. The EEG signal was analyzed in the following frequency ranges: Theta (4–7 Hz) and Alpha (8–12 Hz).

In this work, the short-term effect of *PBM* on *EEG* signals and also a long-term effect is observed. The short-term effect is observed using the ratio of the sum of the power in the alpha band after and before the first recording (*r*1-on the first day of the experiment) (the same is done for the second recording (*r*2-on the last day of the experiment)). The calculation is repeated for the theta band. The ratio is defined as follows:

(1) $$R=\frac{\sum_{i=f_{START}}^{f_{END}}\left(\right|f_{i_{a_{STATE}}}{\left|\right)}^{2}}{\sum_{i=f_{START}}^{f_{END}}\left(\right|f_{i_{b_{STATE}}}{\left|\right)}^{2}},$$

where $f_{i}$ is the *FFT* coefficients of the *i*-th spectral component, *a* and *b* are the recording after and before *PBM* stimulation, respectively, *STATE* represents the *OE* or *CE* state in the *r*1 or *r*2 sessions.

The short-term effect is also observed using the *TAR* index and comparing the ratio of the *TAR* index after and before the *r*1 and *r*2.

The long-term effect is observed using the same indices, but looking at the *r*2 after stimulation and the *r*1 before stimulation.

#### 2.6.2. Connectivity Analysis

In this work, we will observe functional connectivity at predefined intervals. We observe the brain network dynamics applying the index of functional connectivity called Complex Pearson Correlation Coefficient (*CPCC*) [36]. The *CPCC* measure can be divided into two components (measures): the absolute value of the complex Pearson correlation coefficient (*absCPCC*), where a zero phase shift affects the outcome, and the imaginary component of the complex Pearson correlation coefficient (*imCPCC*), where a zero phase shift does not affect the outcome. The relationship between the phase locking value (*PLV*) and *absCPCC*, and the weighted phase-lag index (*wPLI*) and *imCPCC* has been demonstrated numerically and analytically [36], and it was found that *PLV* can be replaced by *absCPCC* and *wPLI* by *imCPCC* [36]. *CPCC* is defined as,

(2) $$CPCC\left(x_{a1},x_{a2}\right)=\frac{\sum_{n=1}^{N}x_{a1,n}\cdot x_{a2,n}^{*}}{\sqrt{\sum_{n=1}^{N}{\left|x_{a1,n}\right|}^{2}}\cdot \sqrt{\sum_{n=1}^{N}{\left|x_{a2,n}\right|}^{2}}},$$

where *N* is the number of samples, $x_{a1}$ and $x_{a2}$ are analytical signals given by Hilbert transform, and ${\left\{.\right\}}^{*}$ is the complex conjugate operator. Where $x_{a1}$ and $x_{a2}$ represent analytical signals given by the Hilbert transform, ${\left\{.\right\}}^{*}$ represents the complex conjugate operator, and *N* represents the number of samples.

In what follows, we will use *imCPCC* which is defined as follows [37]:

(3) $$imCPCC_{x_{a1},x_{a2}}=\left|Im\left[r\left(x_{a1},x_{a2}\right)\right]\right|.$$

## 3. Results

### 3.1. Power Spectrum Analysis

According to [16], the indicator for the diagnosis of Alzheimer’s disease is a significant and widespread decrease in the power of the alpha band and, in contrast, a power increase in theta band. Accordingly, the goal of *PBM* stimulation should be to increase the power of the alpha band and decrease the power of the theta band.

**Figure 7:**
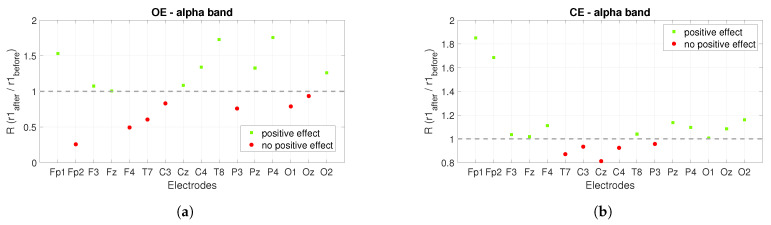

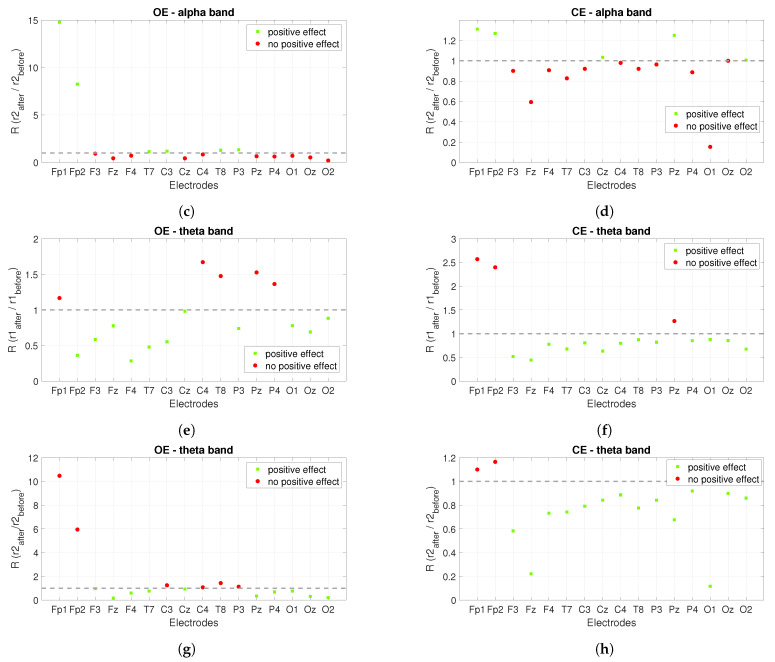
The short-term effect of *PBM* stimulation on the power spectrum when the alpha and theta bands are observed. The figures show the ratios in the observed bands for the *r*1 and *r*2: (**a**,**b**) show the power ratios between the *r*1 after and before stimulation for the alpha band with OE and CE for each electrode, (**c**,**d**) show the ratios for the *r*2. (**e**,**f**) show the power ratios between the *r*1 after and before stimulation for the theta band with *OE* and *CE* for each electrode. Figure (**g**,**h**) show the ratios for the *r*2 for each electrode.

Figure 7 shows the short-term effect of *PBM* stimulation. The ratio of the power of the alpha band after and before each recording was shown, and for the theta band, the power after and before the stimulations was compared. For the alpha band, the ratio is expected to be greater than one, and for the theta band, the aim is for it to be less than one. The green squares mark the ratios for the electrodes that achieved the desired increase in the alpha band and decrease in the theta band, while the red circles mark ratios that are not satisfactory.

For the *r*1, the power ratios for the alpha band under the condition *OE*, showed that there was a visible increase at nine of sixteen electrodes shortly after stimulation (see Figure 7a), and under the condition *CE* at eleven of sixteen electrodes, see Figure 7b. In the *r*2, the positive effect is visible at six of sixteen electrodes for *OE* (Figure 7c) and at five of sixteen electrodes for *CE*, see Figure 7d. Figure 7e plots the ratio of power in the theta band for the *OE* condition in the *r*1. The positive effect is visible on eleven of sixteen electrodes, while for the *CE* condition the positive effect is visible on thirteen of sixteen electrodes, see Figure 7f. For the *r*2 in the OE condition (Figure 7g), the positive effect is visible on ten of sixteen electrodes and for the *CE* condition, the positive effect is visible on fourteen of sixteen electrodes (Figure 7h).

**Figure 8:**
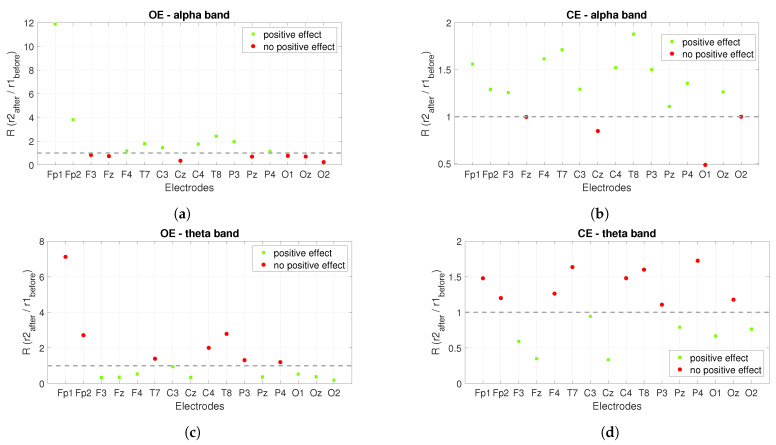
The long-term effect of *PBM* stimulation on the power spectrum when alpha and theta bands are observed. The figures show the power ratios between the *r*2 after stimulation and the *r*1 before stimulation. (**a**) shows the power ratios for the alpha band at *OE*, while Figure (**b**) shows the power ratios at *CE*. (**c**) presents the power ratios for the theta band at *OE*, while (**d**) is for *CE*.

The power ratios between the *r*2 after stimulation and the *r*1 before stimulation show us the long-term effect of *PBM* stimulation, see Figure 8. Similar to Figure 7, a ratio greater than one is expected for the alpha band and the opposite (less than one) for the theta band. The green squares mark satisfactory ratios, while the red circles mark ratios that are not satisfactory. Figure 8a shows the power ratios for the alpha band under the *OE* condition and the positive effect is founded in nine out of sixteen electrodes, while Figure 8b shows the ratios for the alpha band under the *CE* condition and the positive effect is visible in twelve out of sixteen electrodes. The power ratios for the theta band are shown in Figure 8c,d. Figure 8c shows the ratios for the *OE* condition and the positive effect is seen at nine of sixteen electrodes, while for the *CE* condition the positive effect is seen at seven of sixteen electrodes, see Figure 8d.

**Figure 9:**
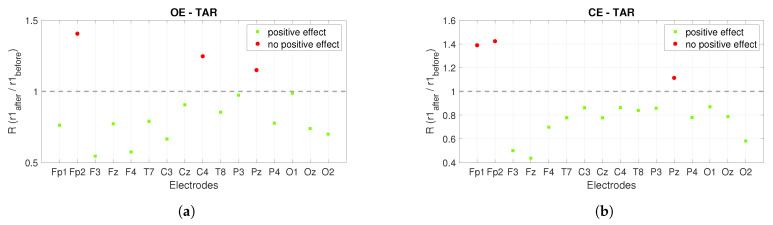

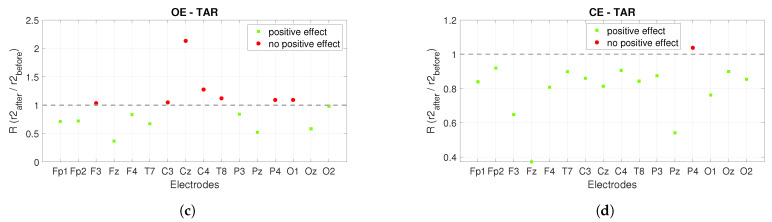
The short-term effect of *PBM* stimulation on the power spectrum is observed using *TAR*. The figures show the ratios of *TAR* for the *r*1 and *r*2 recordings. (**a**) shows the ratios of *TAR* for the *r*1 after and before *PBM* stimulation under the condition *OE*, while (**b**) shows the ratios of *TAR* under the condition *CE*. (**c**,**d**) show the ratios for the *r*2 and *TAR* after and before stimulation was compared.

**Figure 10:**
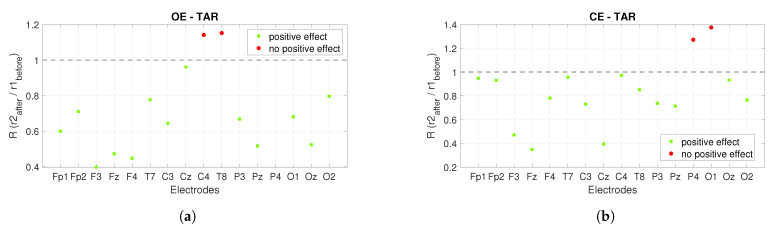
The long-term effect of *PBM* stimulation on the power spectrum is observed using *TAR*. (**a**) shows the ratios of *TAR* between the *r*2 after and the *r*1 before stimulation in the *OE* condition, and (**b**) shows the ratios of *TAR* in the *CE* condition.

Another indicator presented in [16] is *TAR*. It was concluded that a significant and widespread increase in *TAR* is an indicator of Alzheimer’s disease. Therefore, we are expecting to decrease *TAR* by applying *PBM* stimulation. In Figure 9 and Figure 10, the green squares mark the ratios for the electrodes where the *TAR* ratio has reached the desired value (less than one), while the red circles mark the ratios where the desired value is not reached (greater than one).

Figure 9 shows the short-term effect of *PBM* stimulation using *TAR*. Figure 9a shows the ratios of *TAR* between the *r*1 after and before stimulation under the *OE* condition and Figure 9b under the *CE* condition. The positive effect of *PBM* stimulation is visible at thirteen of sixteen electrodes for the conditions *OE* and for *CE*. Figure 9c shows the ratios of *TAR* between the *r*2 after and before stimulation under *OE* and Figure 9d under *CE*. Under the *OE* condition, a positive effect is detected at nine of sixteen electrodes, whereas under the *CE* condition, the positive effect is seen at fifteen of sixteen electrodes.

Figure 10 shows the long-term effects of *PBM* stimulation using *TAR*. The ratios of *TAR* shown in Figure 10 are calculated between the *r*2 after stimulation and the *r*1 before stimulation. Figure 10a shows the observed ratios during the *OE* condition and the positive effect is seen at thirteen of sixteen electrodes, while Figure 10b shows the observed ratios during the *CE* condition and the positive effect is seen at fourteen of sixteen electrodes.

In addition, the electrodes show different positive long-term effects under different eye conditions because brain regions are differently active in different conditions, which is confirmed in [38].

### 3.2. Connectivity Analysis

**Figure 11:**
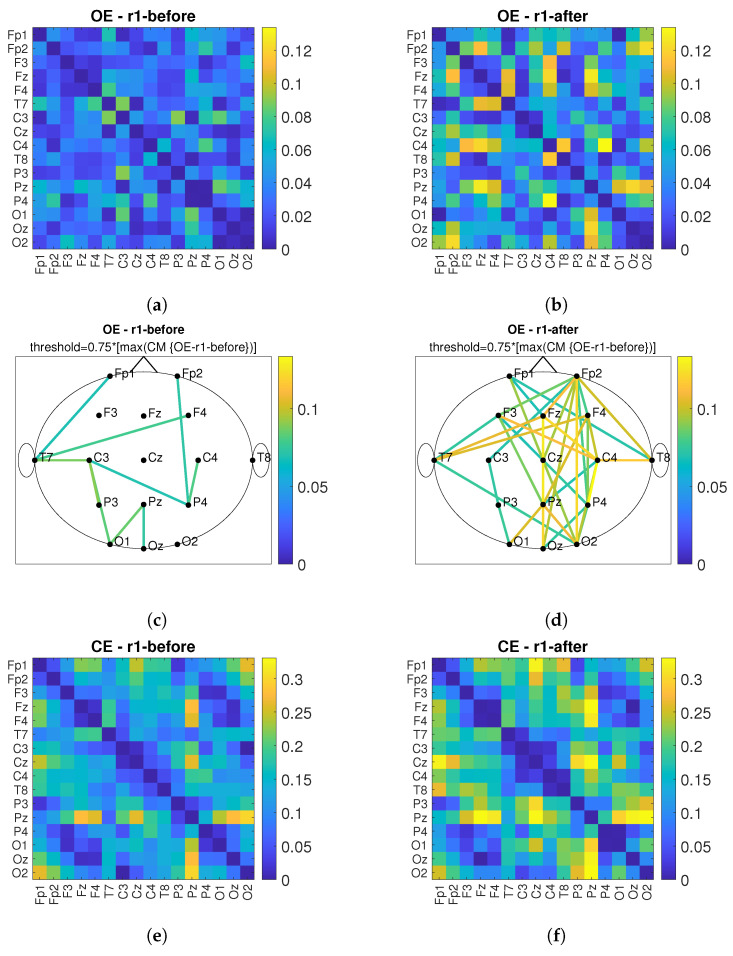

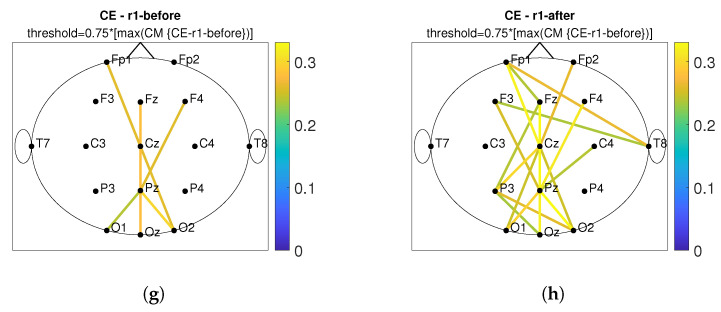
The short-term effect of *PBM* stimulation in observing the connectivity values of *imCPCC* at the *r*1. The connectivity matrices (*CM*) and the head plot of the connections above threshold between electrode pairs are observed. (**a**) shows the *CM* under the *OE* condition before stimulation and (**c**) shows the headplot of the connections, while (**b**,**d**) show the *CM* and the headplot of the connections after stimulation. (**e**–**h**) show the *CM* or the headplot of the *CE* condition.

**Figure 12:**
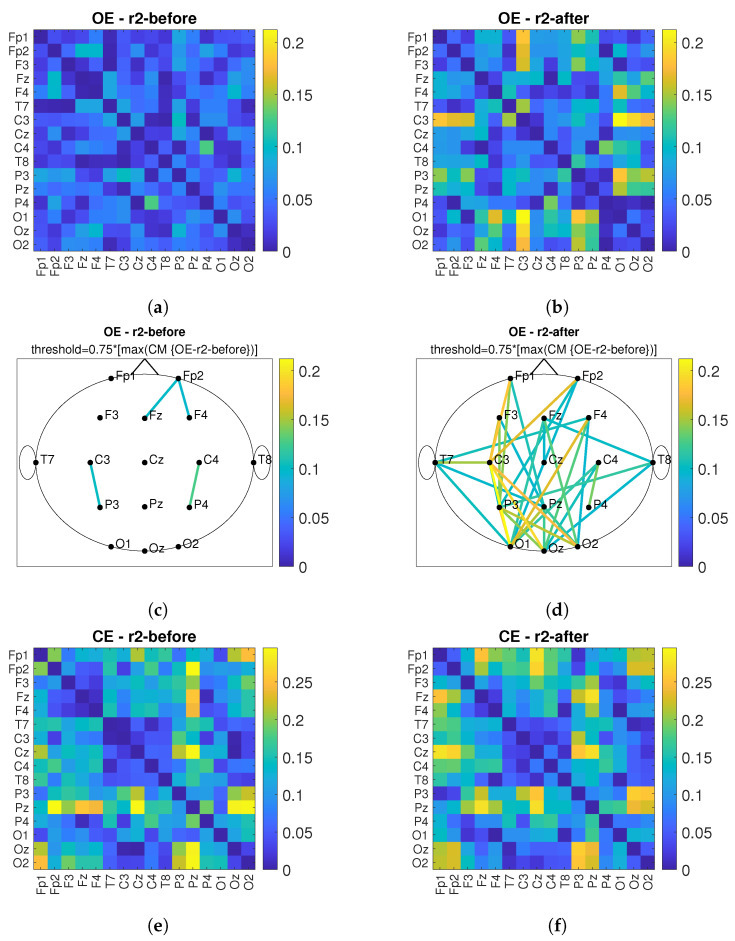

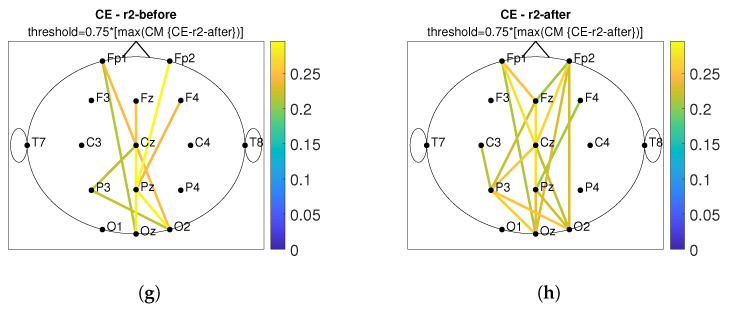
The short-term effect of *PBM* stimulation in observing the connectivity values of *imCPCC* at the *r*2. The connectivity matrices (*CM*) and the head plot of the connections above threshold between electrode pairs are observed. (**a**) shows the *CM* under the *OE* condition before stimulation and (**c**) shows the headplot of the connections, while (**b**,**d**) show the *CM* and the headplot of the connections after stimulation. (**e**–**h**) show the *CM* or the headplot of the *CE* condition.

**Figure 13:**
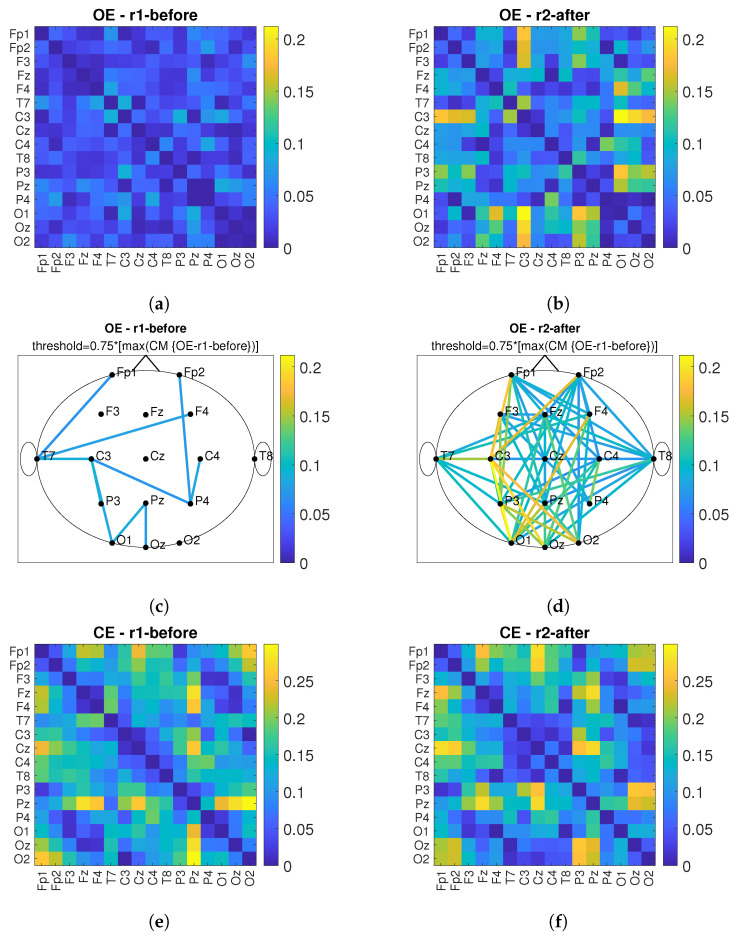

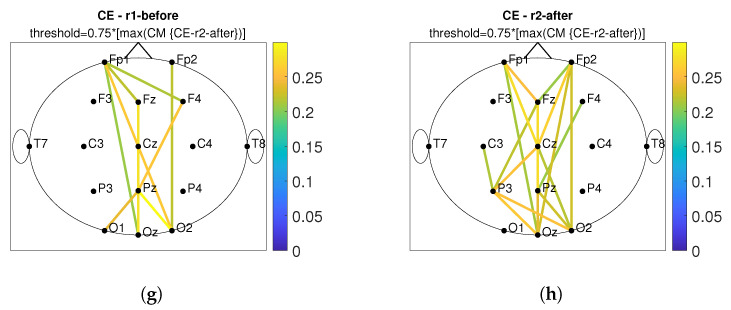
The long-term effect of *PBM* stimulation in observing the connectivity values of *imCPCC* comparing the *r*1 before and the *r*2 after stimulation. The connectivity matrices (*CM*) and the head plot of the connections above threshold between electrode pairs are observed. (**a**) shows the *CM* under the *OE* condition for the *r*1 before stimulation and (**c**) shows the headplot of the connections, while (**b**,**d**) show the *CM* and the headplot of the connections for the *r*2 after stimulation. (**e**–**h**) show the *CM* or headplot of the *CE* condition.

According to [13], connectivity between near and far electrodes for the alpha band decreases in different types of dementia. Therefore, the goal of *PBM* stimulation is to increase the number of connections and connectivity values between the observed electrodes after *PBM* stimulation. Figure 11 shows the connectivity matrices (*CM*) and the head plot of the connections above threshold between pairs of electrodes for the conditions *OE* and *CE* and before/after stimulation. First, the maximum value of the compared *CMs* before and after is determined and the matrix with the lower maximum value is used as the reference matrix. The threshold was set at 75% of the maximum value of the reference matrix. All connections above the threshold are shown in the head plots. The short-term effect is observed and the comparison before and after stimulation is performed for the *r*1, see Figure 11. By comparing Figure 11a,c with Figure 11b,d, it can be observed that the values of connections between electrode pairs are higher after *PBM* stimulation and that the number of electrode pairs increases. The same conclusion is obtained when considering the *CE* condition, which is shown in Figure 11e,g (before stimulation) and Figure 11f,h (after stimulation). Figure 12 shows the short-term effect of *PBM* stimulation for the *r*2. For the conditions of *OE* (Figure 12a–d), it was found that the connection between different brain regions increased more after stimulation compared with *CE* (Figure 12e–h). Figure 13 shows the long-term effects of *PBM* stimulation. A comparison of connectivity between the *r*1 before stimulation and the *r*2 after stimulation for the *OE* (Figure 13a–d) and *CE* (Figure 13e–h) conditions is shown. Higher values of the connection between the electrodes are visible.

Global efficiency was calculated for the *CM* headplots shown in Figure 13c,d,g,h. The global efficiency is defined as follows [39]:

(4) $$E=\frac{1}{n}\sum_{i\in N}E_{i}=\frac{1}{n}\sum_{i\in N}\frac{\sum_{j\in N,j\neq i}d_{ij}^{-1}}{n-1},$$

where $E_{i}$ is the efficiency of node *i*, $d_{ij}$ is the shortest path length (distance) between nodes *i* and *j*, *N* is the set of all nodes in the network, and *n* is the number of nodes. Global efficiency is a measure of the efficiency of remote information transmission in a network. It can be described as the inverse of the average characteristic path length between all nodes in the network [40].

The global efficiency values for all observed *CM*-s can be found in Table 2.

From Table 2, we can conclude that global efficiency increased after treatment with *PBM* stimulation in both conditions with *OE* and *CE*.

## 4. Discussion

In this work, we used a stimulation frequency of 10Hz with power levels 2 and 3 producing a power density of 4.2 and 12.7 mW/cm${}^{2}$ for each of the 3 clusters of *LED*, see Table 1. We chose a stimulation frequency of 10 Hz because the features of Alzheimer’s disease in *EEG* have been described in [16,17,18], where it is confirmed that participants with *AD* showed a significant decrease in spectral and coherence analysis in the alpha band. During the first week of stimulation, three sessions were conducted at power level 2, while during the rest of the time, five sessions per week were conducted at power level 3.

When analyzing the power spectrum, an *FFT* with time segments of 512 samples (1 second) was performed using the Hamming window function. Looking at the power spectrum of the alpha band, we can observe an increase in the power spectrum at most electrodes, observing both the short-term and long-term effect (for the short-term effect, we compare the effect of *PBM* by comparing the power spectrum before and after stimulation for the *r*1 and *r*2 *EEG* recordings, in contrast, the long-term effect was calculated by looking at *r*1 before stimulation and *r*2 (recording after *35* days of *PBM* stimulation) after stimulation). This is a positive effect due to the fact that participants with *AD* usually show a decrease in the alpha band power spectrum [16].

Similar results were reported in [30], where spectral analysis was performed using the Welch method (with a window length of 512 points, *FFT* length of 1024 points, without overlap). They used stimulation at a frequency of 40Hz. The increase of alpha band power spectrum after active *tPBM* was found in [30]. In [30], all frequency bands were observed, but it was emphasized that the changes were greatest in the alpha band. They present the changes in the power spectrum in such a way that they averaged the power spectrum across all electrodes. In this article we show the changes for all electrodes used.

Moreover, similar results are confirmed in [31]. In [31], transcranial infrared laser stimulation (*TILS*) was performed once a week for a period of 5 weeks. The laser wave was continuous and with a power density of 250 mW/cm${}^{2}$. The long-term effect of *TILS* on resting state *EEG* was observed and the effect of *TILS* was found in both left and right hemispheres. The largest increase in the analyzed power spectrum was found in the alpha band both during and after *TILS*. The largest effect was found in the occipital region and the smallest in the parietal and frontal regions.

In [12], the increase in the theta band is presented as an indicator of the detection of *AD*. For this reason, we decided to analyze the power spectrum of the theta band. The aim was to decrease the values of the theta power spectrum by *PBM* stimulation. Considering the short-term effect of the power spectrum in the theta band, a positive effect was found in eleven of sixteen electrodes for *OE* and in thirteen of sixteen electrodes for *CE* in the *r*1, while it was found in ten of sixteen electrodes for *OE* and in fourteen of sixteen electrodes in the *CE* condition in the *r*2. In the theta band, a positive long-term effect was found in nine of sixteen electrodes for *OE* and in seven of sixteen electrodes for *CE*. In [16], it was found that a significant and widespread increase in *TAR* is an important indicator of Alzheimer’s disease. When considering *TAR*, a decrease in *TAR* was considered a positive effect. When considering the short-term effect with the indicator *TAR*, the positive effect in the alpha band was found in thirteen of sixteen electrodes for *OE* and *CE* on the *r*1 and in nine of sixteen electrodes for *OE* and fifteen of sixteen electrodes for *CE* on the *r*2. The positive long-term effect is found at thirteen of sixteen electrodes for *OE* and at fourteen of sixteen electrodes for *CE*.

In this work, we have chosen to use the connectivity measure *imCPCC* which was introduced in [36,37] and could replace *wPLI* for brain connectivity analysis. To evaluate the properties of the brain network (i.e., the network representing the connections between different brain areas), we used the graph-theoretic measure of global efficiency. The alpha band was observed according to [16]. We desired an increase in global efficiency after *PBM* stimulation, which we obtained. Similar results can be found in [30]. There, the influence of active *tPBM* was analyzed using the *wPLI* connectivity measure. To evaluate the integration and segregation properties of the brain network, graph theoretic measures were used, characteristic path length, clustering coefficient, local efficiency, and global efficiency, and the changes were most pronounced in the alpha frequency band for all measures used. The increase in global efficiency was highlighted as a positive effect of *PBM* [30]. In addition, an increase in connectivity in the posterior region was found in [33] using functional magnetic resonance imaging.

## 5. Conclusions

In this article, the effects of stimulation treatment with photobiomodulation are analyzed, a case study is performed. Power spectrum analyses and connectivity analyses are performed. Alpha, theta bands, and *TAR* were observed in the power spectrum analysis, whereas the alpha band was observed in the connectivity analysis. The near-infrared device prototype was designed and realized and *PBM* stimulation was performed in an elderly person with a *SAGE* score indicating probable memory and thinking disorder. The short-term effect and long-term effect of *PBM* stimulation on the power spectrum were analyzed. A positive influence was found in a larger number of electrodes. A connectivity analysis using the *imCPCC* index was also performed. A long-term effect was observed in the calculation of the global efficiency measure. A global efficiency measure was calculated based on connectivity matrices with threshold values. The global efficiency calculated for the long-term effect was higher than before stimulation by a factor of 5.24 in the *OE* condition and by a factor of 1.25 in the *CE* condition. In future work, we will conduct the study with a larger number of participants.

## Figures and Tables

**Table 1:** Mean value of measurements for *LEDs*, power (*P*) in mW and power density ($P_{g}$) in mW/cm${}^{2}$, for 3 diodes in a cluster. Square modulation frequencies of 10 and 40 Hz and continuous generation (*DC*) were tested for different power levels (*PL*).

Type/Measure	10 Hz/PL1	10 Hz/PL2	10 Hz/PL3	10 Hz/PL4	40 Hz/PL2	40 Hz/PL4	DC/PL4
*P*(mW)	1	3.3	10	21.0	3.9	21.0	39
$P_{g}$ (mW/cm${}^{2})$	1.3	4.2	12.7	26.8	5.0	26.8	49.7

**Table 2:** This table contains the values of global efficiency (*E*) calculated for CM-s, which are shown in headplots Figure 13c,d,g,h.

Recordings	*E*
*r*1-*OE* condition before stimulation	0.017
*r*2-*OE* condition after stimulation	0.089
*r*1-*CE* condition before stimulation	0.049
*r*2-*CE* condition after stimulation	0.061

## Abbreviations

The following abbreviations are used in this manuscript:

absCPCC	absolute value of complex Pearson correlation coefficient
AD	Alzheimer’s disease
CE	closed eyes
CM	connectivity matrix
CPCC	Complex Pearson correlation coefficient
CT	Computed tomography
DBS	deep brain stimulation
EEG	Electroencephalography
FDA	U.S. Food and Drug Administration
FFT	Fast Fourier transform
GFS	Global Field Synchronization
IR LED	infra-red Light Emitting Diode
imCPCC	imaginary component of complex Pearson correlation coefficient
LED	Light Emitting Diode
MCI	mild cognitive impairment
MEG	Magnetoencephalography
MI	Mutual Information
MMSE	Mini-Mental State Exam
MRI	Magnetic Resonance Imaging
NIR	near-infrared
OE	opened eyes
PBM	photobiomodulation
PLI	Phase Lag Index
PLV	Phase Locking Value
r1	First Recording
r2	Second Recording
TAR	theta-alpha ratio
tDCS	transcranial direct current stimulation
TILS	transcranial infrared laser stimulation
TMS	transcranial magnetic stimulation
tPBM	transcranial photobiomodulation
SAGE	Self Administered Gerocognitive Exam
SCI	subjective cognitive impairment
wPLI	Weighted Phase Lag Index
